# Lysophosphatidic Acid Inhibits Simvastatin-Induced Myocytoxicity by Activating LPA Receptor/PKC Pathway

**DOI:** 10.3390/molecules25071529

**Published:** 2020-03-27

**Authors:** Kyung-Jong Won, Yu-Jin Goh, Sung-Hee Hwang

**Affiliations:** 1 Department of Physiology and Medical Science, School of Medicine, Konkuk University, Chungju 27478, Korea; 2 Department of Pharmaceutical Engineering, College of Health Sciences, Sangji University, Wonju 26339, Korea

**Keywords:** lysophosphatidic acid, simvastatin, L6 cells, cytotoxicity, muscle cell

## Abstract

Statins such as simvastatin have many side effects, including muscle damage, which is known to be the most frequent undesirable side effect. Lysophosphatidic acid (LPA), a kind of biolipid, has diverse cellular activities, including cell proliferation, survival, and migration. However, whether LPA affects statin-linked muscle damage has not been reported yet. In the present study, to determine whether LPA might exert potential protective effect on statin-induced myocyotoxicity, the effect of LPA on cytotoxicity in rat L6 myoblasts exposed to simvastatin was explored. Viability and apoptosis of rat L6 myoblasts were detected via 2,3-bis(2-methoxy-4-nitro-5-sulfophenyl)-5- [(phenylamino)carbonyl]-2*H*-tetrazolium hydroxide (XTT) assay and terminal deoxynucleotidyl transferase-mediated dUTP nick end labeling (TUNEL) assay, respectively. Protein expression levels were detected via Western blotting. Simvastatin decreased viability of L6 cells. Such decrease in viability was recovered in the presence of LPA. Treatment with LPA suppressed simvastatin-induced apoptosis in L6 cells. In addition, treatment with LPA receptor inhibitor Ki16425, protein kinase C (PKC) inhibitor GF109203X, or intracellular calcium chelator BAPTA-AM attenuated the recovery effect of LPA on simvastatin-induced L6 cell toxicity. These findings indicate that LPA may inhibit simvastatin-induced toxicity in L6 cells probably by activating the LPA receptor-PKC pathway. Therefore, LPA might have potential as a bioactive molecule to protect muscles against simvastatin-induced myotoxicity.

## 1. Introduction

Statins have excellent antihyperlipidemic activity. Therefore, they have been widely used for treating hyperlipidemia [1,2,3] and decreasing cardiovascular risk [2,4,5]. Statins such as pravastain, fluvastatin, lovastatin, atrovastatin, rosuvastatin, simvastatin, and so on are known to be able to decrease the synthesis of cholesterol by inhibiting 3-hydroxy-3-methylglutaryl coenzyme A (HMG CoA) reductase, a determinant enzyme for synthesizing mevalonate from HMG CoA which is one of the most important steps in the biosynthesis of cholesterol [3,6]. HMG CoA is necessary to make cholesterol. It is also needed for the synthesis of other molecules such as CoQ10 (ubiquinone) and selenoproteins that play an important role in antioxidative defense and ATP synthesis [6,7,8]. Statins can induce reactive oxidative stress that could result in cell death [9,10,11]. These reactions caused by statins are known to be related to their side effects including liver toxicity and myopathy [6,9,10,11]. Statins are usually well tolerated by most people [1,2,3]. However, some people are intolerant to statins. They may experience clinical adverse effects such as hepatotoxicity, diabetes mellitus, and skeletal muscle toxicity [11,12,13,14,15,16]. It has been reported that high-dosage statin can cause muscle cytotoxicity-related symptoms including muscle pain, muscle weakness, rhabdomyolysis, and myositis [14]. One of the most frequent adverse effects of statins is muscle related adverse effect which is the main cause of discontinuation of statin uses [3,14,16].

Lysophosphatidic acid (LPA), which is one of the glycerophospholipids with one fatty acid chain and a phosphate, has been found in various mammalian cells [17]. It is biologically synthesized from other lysophospholipids like lysophosphatidylcholine by lysophospholipase D (autotaxin) or from phosphatidic acid by the action of phospholipase A1 [17]. LPA is known as a potent signaling molecule with biological effects. It is also a ligand of LPA receptors belonging to G-protein coupled receptors [17,18]. Through activation of LPA receptors, LPA can trigger cell proliferation, cell migration, cell survival, and intracellular calcium mobilization in many cell types [17,18,19,20]. Recent studies have revealed that LPA signaling might be involved in hair follicle development, vascular development, lymphatic development, and reproduction [17,18,19]. Dysregulation of LPA receptor signaling can cause some pathological conditions such as neuropathy, cardiovascular disease, inflammation, infertility, and cancers [20]. LPA can also enhance the proliferation of skeletal muscle cells, although this is controversial [21,22,23,24,25]. It has been shown that LPA can stimulate the growth of mouse C2C12 myoblast cells via phosphoinositide-3-kinase (PI3K)-dependent pathway and intracellular calcium increase [21,23,24] with endogenous monocyte chemotactic protein-1 (MCP-1) expression and secretion [25]. However, Baptiste et al. [22] have shown that C2C12 cells express LPA receptors and that LPA can mediate the activation of extracellular signal-regulated protein kinases 1 and 2 (ERK1/2) mitogen-activated protein (MAP) kinase and Akt/protein kinase B (PKB) known as mitogenic signaling molecules, although it cannot induce proliferation of C2C12 cells. These reports imply that the biological effect of LPA and its-related signaling pathway may need to be clarified in skeletal muscle cells.

There have been many attempts to reduce side effects of statins [1,16,26,27]. For example, reducing statin dose and switching to non-statin-based lipid-lowering therapies like ezetimibe have been tried [1,16,26,27]. The combination therapy of ezetimibe with simvastatin can result in more beneficial effects than simvastatin monotherapy for patients [1,16,26,27]. Moreover, LPA has also known as survival factors showing anti-apoptotic effects in many different cell types including cancer cells, fibroblasts, macrophages [18,28]. LPA has shown to attenuate statin-induced apoptosis in vascular endothelial cells [29]. Although studies have been attempted on the development of new therapies or bioactive substances to improve the side effects of statins [30,31], the role of LPA in statins-induced myotoxicity has not been reported yet. Thus, the present study hypothesized that LPA might protect skeletal muscle cells from statin-induced myotoxicity. To test this hypothesis, we examined potential myocytotoxic effects of simvastatin on L6 cells from rat skeletal muscle and defined the inhibitory effect of LPA, a bioactive lipid, on simvastatin-induced cytotocixity in L6 cells.

## 2. Results

### 2.1. Effect of LPA on Simvastatin-Induced Cytotoxicity in L6 Cells

**Figure 1:**
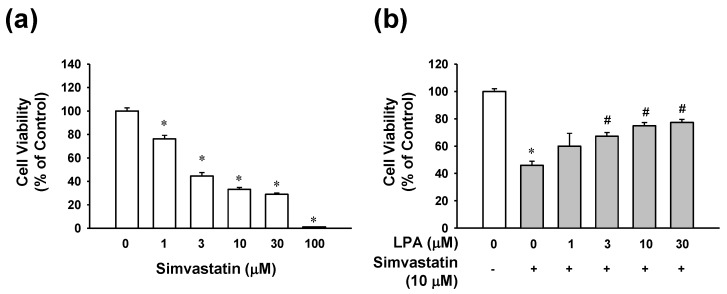
Effect of lysophosphatidic acid on viability of simvastatin-treated L6 cells. (**a**) Effect of simvastatin on L6 cell viability. Cells were incubated with simvastatin (1–100 μM) in serum free medium for 48 h. (**b**) Effect of lysophosphatidic acid (LPA) on L6 cell viability in the prescence of simvastatin. L6 cells were treated with LPA (1–30 μM) in the presence of simvastatin (10 μM) for 48 h. Cell viability was analyzed using an XTT assay. Cell viability level of untreated control was considered as 100%. Data represent the means ± SEM (n = 6). * *p* < 0.05 vs. untreated control; # *p* < 0.05 vs. simvastatin alone.

To determine the potential role of LPA in myocytotoxicity in the presence of simvastatin, the effect of simvastatin on L6 cell viability was first analyzed using the 2,3,-bis(2-methoxy-4-nitro-5-sulfophenyl)-5-[(phenylamino)-carbonyl]-2*H*-tetrazolium) (XTT) assay. As shown in Figure 1a, simvastatin (1–100 μM) reduced the viability of L6 cells in a dose-dependent manner. Next, the effect of LPA on simvastatin-reduced L6 cell viability was examined in L6 cells treated with different concentrations of LPA in the presence of 10 μM of simvastatin. In the presence of 10 μM of simvastatin, treatment with LPA at 1–30 μM dose-dependently increased the viability of L6 cells. Its effect was significant at 3–30 μM, reaching a plateau at 10 μM (Figure 1b). In addition, treatment with LPA alone did not influence cell viability of L6 cells (data not shown).

### 2.2. Effect of LPA on Simvastatin-Induced Apoptotic Cell Death in L6 Cells

**Figure 2:**
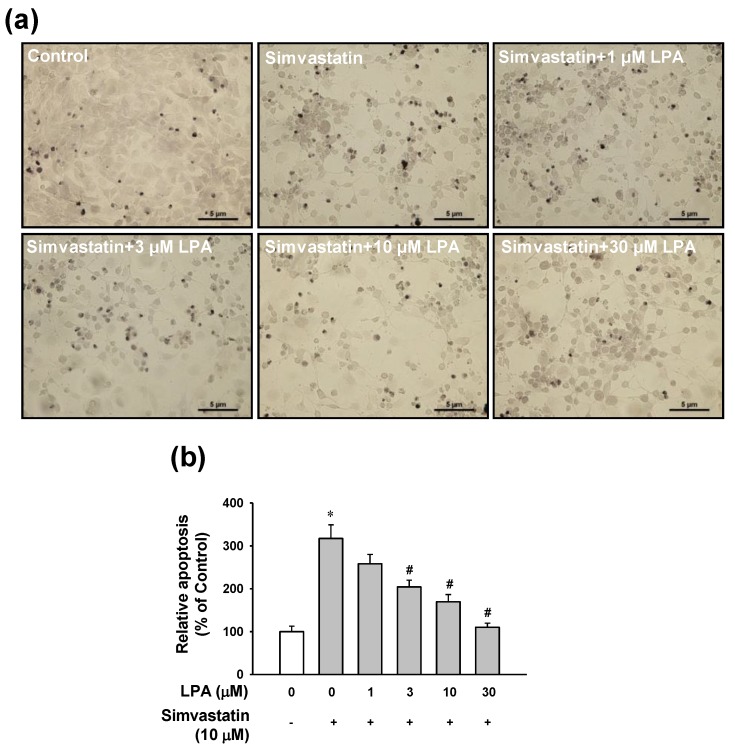
Effect of LPA on simvastatin-induced apoptosis in L6 cells. (**a**) Representative images obtained by each treatment. Cells were cultured on a cover glass in a 24-well plate and incubated with LPA (0–30 μM) in the presence of simvastatin (10 μM) in serum free medium for 48 h, Apoptosis of cells was measured using a TUNEL assay. (**b**) Statistical graph obtained from panel (a). Brown spots indicate TUNEL-positive cells. Level of untreated control was considered as 100%. Data represent the means ± SEM (n = 6). * *p* < 0.05 vs. untreated control; # *p* < 0.05 vs. simvastatin alone.

To examine whether the protective effect of LPA on simvastatin-induced cytotoxicity was associated with anti-apoptotic events, apoptosis level of LPA-treated cells in the presence of simvastatin was determined by a terminal deoxynucleotidyl transferase (TdT)-mediated dUTP nick end-labeling (TUNEL) assay. As shown in Figure 2, simvastatin (10 μM) increased the number of apoptotic cells by 3-fold compared with the untreated control group.

LPA (1–30 μM) reduced the number of simvastatin (10 μM)-induced apoptotic cells in a dose-dependent manner. LPA at 30 μM almost completely recovered the apoptotic level induced by simvastatin (10 μM) (Figure 2).

### 2.3. Effect of LPA on Caspase-3 and BAX Signals in L6 Cells Treated with Simvastain

**Figure 3:**
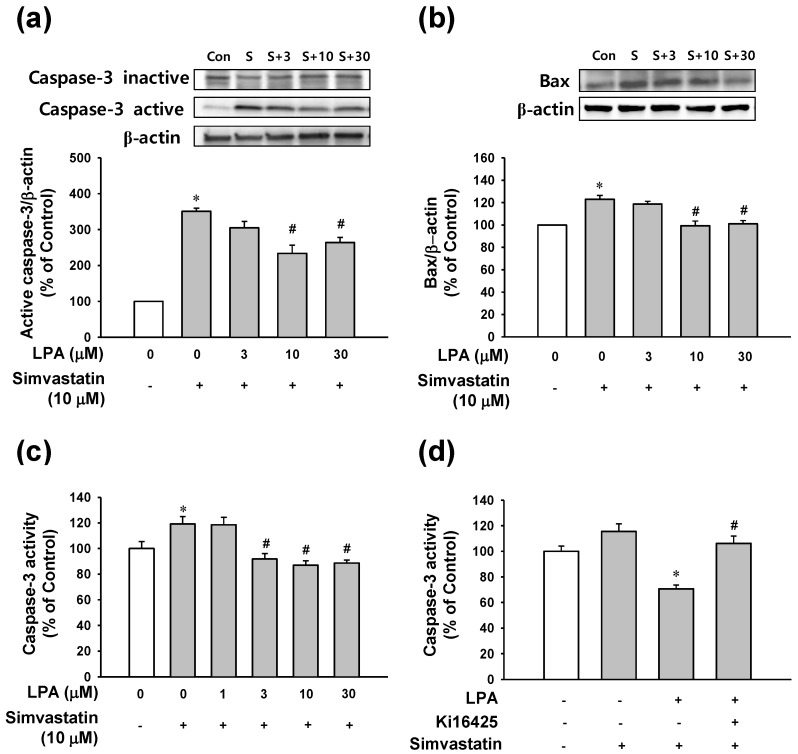
Effect of LPA on apoptosis-related proteins in simvastatin-treated L6 cells. Effect of LPA on the simvastatin-induced caspase-3 activation (**a**) and Bax expression (**b**) in L6 cells. Cells were serum-starved for 6 h and then treated with LPA (0–30 μM) in the presence of simvastatin (10 μM) in serum free medium for 48 h. Cell lysates were subjected to immunoblotting with the indicated antibodies (n = 4 for each antibody) (**a**,**b**). (**c**,**d**) Effect of LPA on simvastatin-induced caspase-3 activity (**c**) and effect of LPA receptor inhibitor on LPA-reduced caspase-3 activity (**d**) in L6 cells. Cells were treated with LPA at indicated concentrations in the presence of 10 μM of simvastatin for 5 h (n = 6) (**c**). Cells were incubated with or without LPA receptor inhibitor Ki16425 (5 μM) in the presence of LPA (10 μM) or simvastatin (10 μM) in serum free medium for 5 h (n = 6) (**d**). Cellular caspase-3 activity was measured as mentioned in the Materials and Methods section. Data represent the means ± SEM. Levels in untreated controls were considered as 100%. (a–c) * *p* < 0.05 vs. untreated control; # *p* < 0.05 vs. simvastatin alone. (**d**) * *p* < 0.05 vs. simvastatin alone; # *p* < 0.05 vs. LPA with simvastatin. Immunoblot bands: S, simvastatin (10 μM); S+3, simvastatin (10 μM)+LPA (3 μM); S+10, simvastatin (10 μM)+LPA (10 μM); S+30, simvastatin (10 μM)+LPA (30 μM).

The anti-apoptotic effect of LPA mentioned above was confirmed by analyzing expression levels of caspase-3 and Bax as apoptosis-related proteins using an immunoblotting technique and caspase-3 activity using an immunosorbent enzyme assay. As shown in Figure 3a, simvastatin (10 μM) elevated the level of cleaved caspase-3 (active form) and reduced the level of pro-caspase-3 (inactive form). LPA at 10 and 30 μM significantly reduced simvastatin (10 μM)-cleaved caspase-3 level. Bax expression level increased by simvastatin (10 μM) was also attenuated by treatment with LPA at 10 and 30 μM (Figure 3b). In addition, simvastatin (10 μM) significantly increased caspase-3 activity. Such increase was significantly reduced by treatment with LPA at 3–30 μM (Figure 3c). Furthermore, LPA (10 μM)-reduced simvastatin (10 μM)-increased caspase-3 activity in L6 cells; this effect was attenuated by treatment with Ki16425 (5 μM), an inhibitor of LPA receptor (Figure 3d).

### 2.4. Involvement of LPA Receptor and PKC in the Protective Effect of LPA on Simvastatin-Induced Cytotoxicity

**Figure 4:**
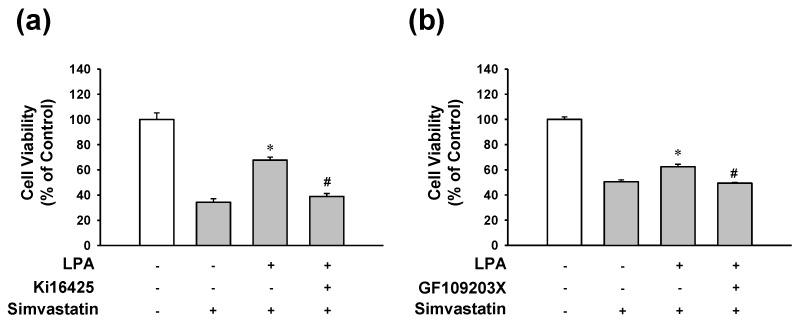
Effects of LPA or PKC inhibition on the viability of LPA-treated L6 cells in the presence of simvastatin. Cells were incubated with or without LPA receptor inhibitor Ki16425 (5 μM) (**a**), or PKC inhibitor GF109203X (1 μM) (**b**) in the presence of LPA (10 μM) or simvastatin (10 μM) in serum free medium for 48 h. Cell viability was measured using an XTT assay. Levels in the untreated control were considered as 100%. Data represent the means ± SEM (n = 6 for each experiment). * *p* < 0.05 vs. simvastatin alone; # *p* < 0.05 vs. LPA with simvastatin.

To determine signaling pathways associated with LPA’s action on L6 cell response in the presence of simvastatin, the effect of LPA on simvastatin-induced L6 cell toxicity was tested in the presence or absence of LPA receptor inhibitor Ki16425 or PKC inhibitor GF109203X. LPA (10 μM)-induced inhibition of L6 cell viability reduction in the presence of simvastatin was completely blocked by treatment with Ki16425 (5 μM) (Figure 4a). Treatment with GF109203X (1 μM) showed similar pattern to the effect of Ki16425 (5 μM) on LPA and simvastatin-induced cell viability (Figure 4b).

### 2.5. Involvement of MAP Kinase and Intracellular Calcium Mobilization in the Protective Effect of LPA on Simvastatin-Induced Cytotoxicity

**Figure 5:**
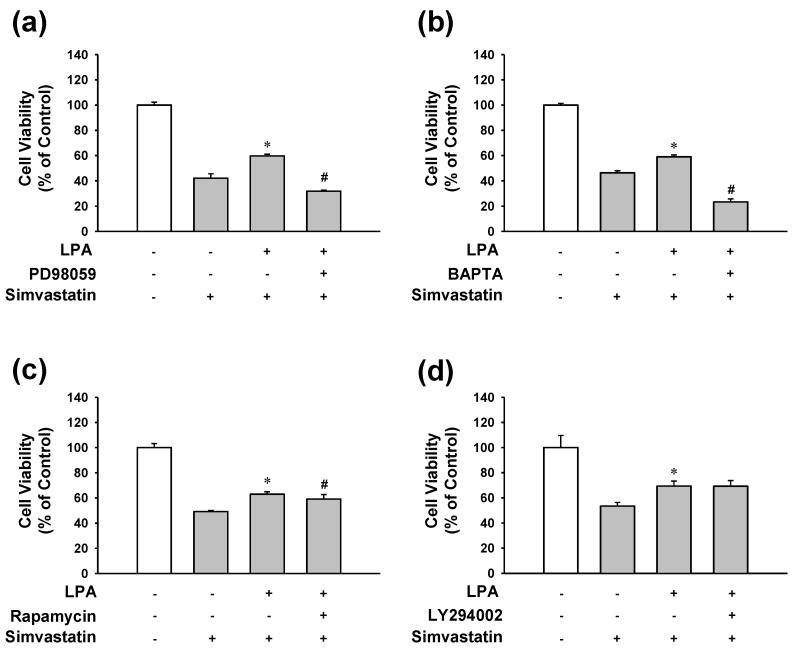
Effects of MAP kinase, intracellular calcium, PI3K, or mTOR inhibition on the viability of LPA-treated L6 cells in the presence of simvastatin. Cells were treated with or without ERK1/2 inhibitor PD98059 (10 μM) (**a**), intracellular calcium chelator BAPTA-AM (5 μM) (**b**), PI3K inhibitor LY294002 (25 μM) (**c**), or mTOR inhibitor rapamycin (2 μM) (**d**) in the presence of LPA (10 μM) or simvastatin (10 μM) in serum free medium. After 48 h incubation, XTT assay was performed. Data represent the means ± SEM (n = 6 for each experiment). * *p* < 0.05 vs. simvastatin alone; # *p* < 0.05 vs. LPA with simvastatin.

It is known that LPA receptor activation can lead to various downstream signaling pathways depending on cell types, including MAP kinase (ERK1/2) activation and transient increase in intracellular calcium concentration, PI3K and AKT, and mTOR activation [18,19,32]. Thus, downstream signaling pathways linked to LPA receptor activation in simvastatin-treated L6 cells were determined by LPA treatment with or without several inhibitors. The inhibitory effect of LPA on simvastatin-induced decrease of L6 cell viability was completely blocked by MAP kinase ERK1/2 inhibitor PD98059 (10 μM) (Figure 5a) or by cell permeable calcium chelator 1,2-Bis(2-aminophenoxy) ethane-*N*,*N*,*N*′,*N*′-tetraacetic acid tetrakis(acetoxymethyl ester) (BAPTA-AM) (5 μM) (Figure 5b). In addition, mTOR inhibitor rapamycin (2 μM) slightly but significantly reduced the inhibitory effect of LPA on simvastatin-induced reduction of L6 cell viability (Figure 5c). On the other hand, PI3K inhibitor LY294002 (25 μM) did not significantly affect the inhibitory effect of LPA (Figure 5d).

## 3. Discussion

Statins are very effective for treating patients with hypercholesterolemia. Although statins have improved the mortality of patients with high-risk of cardiovascular diseases [4,5], statin intolerance still remains. In the Prediction of Muscular Risk in Observational Conditions (PRIMO) study, muscle-related symptoms of 7924 patients receiving high-dosage statin therapy in France were examined and muscular symptoms were reported by 10.5% of these patients [14,33]. Muscle symptoms included muscle pain and weakness with or without creatine kinase elevations, myositis, or rhabdomyolysis [14,33]. The occurrence of side effects could result in discontinuation of statins treatment in some patients because there is no effective treatment for statin-induced muscle toxicity [3,14,16]. Therefore, strategies are needed to reduce statin-associated adverse events in muscle symptoms.

Many studies have demonstrated that LPA can act as a cell proliferation-inducing growth factor by activating LPA receptor and downstream signaling pathways in various cell types, including ovarian cancer cells, neuronal cells, smooth muscle cells, and B-cells [28,34]. As similar to other growth factors, LPA can also act as a survival factor by protecting many cell types (including fibroblast, immune cells, neuronal cells, and cardiac myocytes) against apoptosis [18,28]. Moreover, it has also been reported that LPA pretreatment prevents statin-induced endothelial cell apoptosis [29]. Statins have also been shown to reduce LPA-induced proliferation of smooth muscle cells [35]. Although these reports have used cell types different from the cell type used in our study, it can be assumed that LPA may protect L6 myoblasts from simvastatin-induced cytotoxicity. In the present study, we found that LPA significantly increased viability of simvastatin-treated L6 cells in a dose-dependent manner, indicating that LPA might be able to improve simvastatin-induced cytotoxicity of skeletal muscle cells. Simvastatin-induced apoptosis was inhibited by treatment with LPA. Increased expression levels of cleaved caspase-3 and Bax as apoptosis markers in L6 cells by simvastatin treatment were attenuated by treatment with LPA. Immunosorbent enzyme assay also showed that simvastatin-induced caspase-3 activity in L6 cells was reduced in the presence of LPA. These results imply that simvastatin may induce L6 cell toxicity and that LPA may exert a protective effect by inhibiting apoptotic activity.

**Figure 6:**
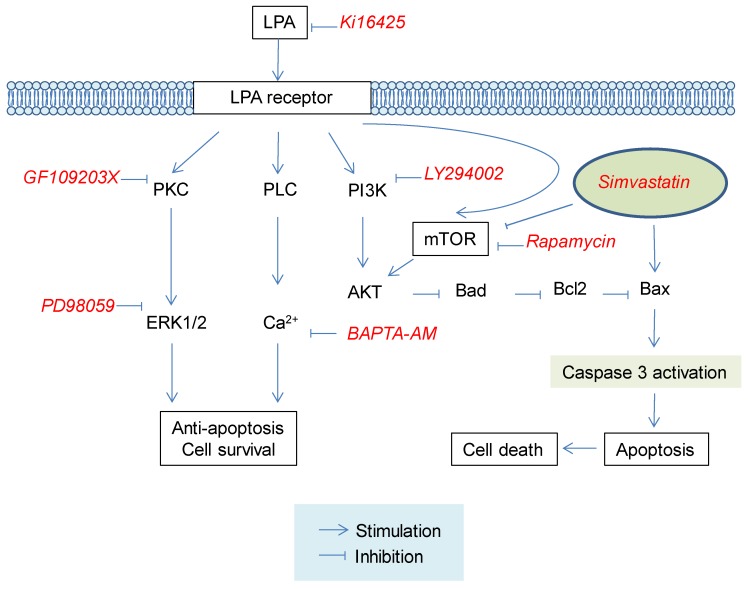
Proposed signaling pathways for the protective effect of LPA against simvastatin-induced cytotoxicity in L6 cells. LPA receptor activation by LPA may influence PKC/ERK1/2, PLC/intracellular Ca^2+^ mobilization, and mTOR/AKT pathways, leading to cell survival. Such cell survival effect may result in inhibition of simvastatin-induced cytotoxicity in L6 cells. LPA, lysophosphatidic acid; PKC, protein kinase C; PLC, phospholipase C; ERK1/2, Extracellular signal-regulated kinase 1/2; mTOR, mammalian target of rapamycin; PI3K, Phosphoinositide 3-kinases; Akt, Protein kinase B; Bad, Bcl-2 associated agonist of cell death; Bax, Bcl-2-associated X protein; Bcl-2, B-cell lymphoma 2.

Various responses of LPA receptor are known to be mediated by different G protein-coupling [18,19,20]. Activation of G_i/o_ inhibits adenyl cyclase (AC) and cAMP production, but activates the Ras/MAP kinase cascade [19,36]. In addition, G_i/o_ can activate PLC via its βγ-subunits. Alpha-subunits of G_q_ proteins are primary effectors of PLC activation [19,36]. PLC activation will result in the generation of diacylglycerol (DAG) and inositol triphosphate (IP_3_) from phosphatidylinositiol diphosphate (PIP_2_) [18,19,20]. DAG can activate protein kinase C (PKC). IP_3_ can mobilize [Ca^2+]^_i_ [18,19,20]. G_12/13_ proteins are responsible for the activation of Rho, a small GTPase that can stimulate Rho kinases [19,36]. These reports imply that LPA receptor-related signals might be involved in protective effect of LPA against simvastatin-induced L6 cell cytotoxicity. Several reports have revealed that the protective effect of LPA against apoptosis is exerted by activation of PI3K/AKT signaling pathways in ovarian cancer cell, HeLa cells, and B-cells [33]. Moreover, it has been suggested that the protective effect of LPA against apoptosis of fibroblast cells might be mediated by the MAP kinase signaling pathway [28]. Interestingly, in the present study, we observed that inhibition of LPA receptor, PKC, MAP kinase, or intracellular calcium chelator reduced the inhibitory effect of LPA on simvastatin-induced cytotoxicity, suggesting that the activation of LPA receptor by LPA with subsequent activations of PKC, MAP kinase, and intracellular calcium mobilization might have contributed to the protective effect of LPA against simvastatin-induced cytotoxicity (Figure 6).

Moreover, it has been reported that LPA receptor activation can lead to activation of Rho, PKC, phospholipase D1, and eventually mTOR [32]. Phosphatidic acid produced from LPA by phospholipase D could also stimulate mTOR activity for cell survival [37]. Statins can suppress mTOR and AKT signaling, leading to apoptotic signaling including caspase-3 activation and finally cell death [38]. In the present study, mTOR inhibitor rapamycin reduced the inhibitory effect of LPA on simvastatin-induced cytotoxicity, although PI3K inhibitor LY294002 did not block the protective effect of LPA on simvastatin-induced cytotoxicity. Thus, LPA might indirectly attenuate statin-induced muscle cell toxicity by activating mTOR signals, at least partially.

In summary, the present study demonstrated that simvastatin caused cytotoxicity to L6 cells and that LPA treatment attenuated the simvastatin-induced cytotoxicity. Simvastatin induced apoptosis of L6 cells. Such effect was prevented by LPA. Treatment with LPA receptor inhibitor, PKC inhibitor, ERK inhibitor, or calcium chelator almost completely attenuated the recovery effect of LPA on simvastatin-induced cell toxicity, although mTOR inhibitor also slightly inhibited the protective effect of LPA. These findings indicate that LPA might be able to inhibit simvastatin-induced cytotoxicity in L6 cells probably through activation of LPA receptor-PKC signaling pathway. Therefore, LPA might be a promising bioactive molecule for protecting muscles against simvastatin-induced myocytotoxicity.

## 4. Materials and Methods

### 4.1. Materials

LPA was purchased from Avanti Polar Lipids, Inc. (Alabaster, AL, USA). Dulbecco’s Modified Eagle’s Medium (DMEM), caspase-3 monoclonal antibody (CPP32 4-1-18), and other cell culture reagents were purchased from Thermo Fisher Scientific Korea (Seoul, South Korea). Goat anti-mouse IgG conjugated with horse radish peroxidase was purchased from Santa Cruz Biotechnology, Inc. (Dallas, TX, USA). Goat anti-beta actin monoclonal antibody conjugated with horse radish peroxidase was purchased from Abcam (Cambridge, MA, USA). All other reagents used were purchased from Sigma-Aldrich (St. Louis, MO, USA).

### 4.2. Cell Culture

Rat myoblast L6 cells were purchased from American Type Culture Collection (Manassas, VA, USA). They were cultured in DMEM supplemented with 10% (*v*/*v*) fetal bovine serum (FBS), 100 μg/mL streptomycin, and 100 U/mL penicillin.

### 4.3. Cell Viability

Cell viability was determined with XTT-based assay as previously described [39] with some modifications. Briefly, cells were seeded into 96-well plates at 5 × 10^3^/well. After 24 h, cells were washed with serum-free DMEM. These cells were then incubated with LPA at indicated concentrations with or without simvastatin or inhibitors. In all the experiments of cells incubated with LPA and inhibitors, inhibitors were added to the cells simultaneously with LPA. After 48 h incubation, the culture medium was replaced with 100 μL of serum free medium without phenol red. Twenty-five microliters of XTT reaction solution containing 1 mg/mL XTT and 0.036 mg/mL of phenazine methylsulfate was then added to each well. After 2 h of incubation, absorbance was measured at 450 nm.

### 4.4. TUNEL ASSAY

A TUNEL assay was performed in accordance with the manufacturer’s protocol Roche, Basel, Switzerland). Briefly, L6 cells (3 × 10^4^) were cultured on cover glass in 24-well cell culture plate and treated with LPA at different concentrations in the presence of 10 μM simvastatin for 48 h in a 5% CO_2_ incubator at 37 °C. These cells were then fixed with 4% paraformaldehyde in PBS for 1 h at room temperature (RT). These fixed cells were treated with permeabilization solution (0.1% Triton X-100) for 2 min at RT and then incubated with 50 μL of TUNEL reaction buffer for 1 h in a 37 °C humidified atmosphere in the dark. After incubation, cells were then treated with 50 μL of converter-POD (anti-fluorescein antibody, Fab fragment from sheep, conjugated with horse-radish peroxidase) in a 37 °C humidified chamber for 30 min and then treated with 50 μL DAB (3,3’-diaminobenzidine) substrate for 10 min at RT. Apoptosis was estimated by counting TUNEL-positive brown cells using a BX51 light microscope (Olympus, Tokyo, Japan).

### 4.5. Immunoblotting

Cells were treated with LPA for 48 h with or without simvastatin and lysed with modified RIPA buffer (50 mM Tris-HCl, 150 mM NaCl, 1% NP-40, 0.25% sodium deoxycholate, 1mM EGTA, pH8.0, with protease inhibitors). Caspase-3 and Bax protein expression levels were then determined by sodium dodecyl sulfate polyacrylamide gel electrophoresis and immunoblotting using caspase-3 monoclonal antibody (Invitrogen, Carlsbad, CA, USA) and rabbit monoclonal antibody anti-Bax antibody (Cell signaling, Danvers, MA, USA), respectively. The blotted membrane was stripped and reblotted for beta actin using anti-β-actin monoclonal antibody (Sigma-Aldrich, St. Louis, MO, USA). Data capture and processing were performed with a luminescent image analyzer LAS-4000 and a Multi Gauge software (version 3.0, Fujifilm, Japan).

### 4.6. Caspase-3 Activity Assay

Cells were seeded into 96-well plates at 5 × 10^3^/well. After 24 h, cells were washed with serum-free DMEM and starved for 6 h. These cells were treated with LPA at different concentrations in the presence of 10 μM of simvastain. After 5 h of incubation, cellular caspase-3 activity was measured using a caspase-3 assay kit, Fluorimetric (Sigma-Aldrich, St. Louis, MO, USA), according to the manufacturer’s instruction. Fluorescence signal produced from hydrolysis of the fluorescent 7-amino-4-methylcoumarin (AMC) by caspase-3 was measured using a fluorescence microplate reader (Gemini EM, Molecular Devices, San Jose, CA, USA) with an excitation wavelength of 360 nm and an emission wavelength of 460 nm. Caspase-3 activity was then calculated from the standard curve of AMC.

### 4.7. Statistical Analysis

Data are expressed as means ± standard error of the mean (SEM). Statistical analysis of data was performed using Student’s *t*-test or one-way analysis of variance (ANOVA) followed by Bonferroni post-hoc test for multiple comparisons. All statistical evaluations were performed using GraphPad prism, version 5.0 (Graph Pad Software, San Diego, CA, USA). *p*-Values less than 0.05 were considered statistically significant.
